# Home environment as a factor in maintaining the mental health of the individual in the family

**DOI:** 10.1192/j.eurpsy.2021.399

**Published:** 2021-08-13

**Authors:** E. Gutkevich, E. Shalygina, Y. Maltseva, S. Vladimirova

**Affiliations:** 1 Psychology Department, National Research Tomsk State University, Tomsk, Russian Federation; 2 Endogenous Disorders Department, Mental Health Research Institute, Tomsk, Russian Federation; 3 Department Of Coordination Of Research, Mental Health Research Institute, Tomsk National Research Medical Center, Russian Academy of Sciences, Tomsk, Russian Federation

**Keywords:** family, mental patients, readmission

## Abstract

**Introduction:**

The relevance of studying the characteristics of the home environment of a person with mental health problems is determined by the need to identify the resources of the individual and the family to form multilevel adaptive competencies aimed at maintaining mental health.

**Objectives:**

The present study was conducted to obtain standardized assessments of attitudes towards the home environment, towards the home as a place of functioning of the family with mental patients.

**Methods:**

The study involved 12 patients aged 21-60 years diagnosed according to ICD-10 F2 with the disease duration of more than 1 year and readmission. Methods used were experimental psychological questionnaire “My home” (Reznichenko, Nartova-Bochaver, Kuznetsova, 2016), mathematical statistics

**Results:**

The test results showed that the average score for the “strength of significance of the home for its inhabitants” across the data set was 4.14, which differed from the average value of 3.73.

**Conclusions:**

The psychological foundations of attachment can be associated with a variety of facts, including the frequent absence of patients outside the home environment during readmission periods. The home environment can be a complex of positive feelings and experiences in relation to the home as a personally significant place. The study revealed some of the psychological traits of the subjective attitude to home, which can become indicators of psychological adaptation in persons with mental disorders in the future. Reznichenko S.I., Nartova-Bochaver S.K., Kuznetsova V.B. (2016) Home Attachment Assessment Method. Psychology. Journal of the Higher School of Economics. 13(3): 498-518.

**Disclosure:**

No significant relationships.

